# Role of Small Envelope Protein in Sustaining the Intracellular and Extracellular Levels of Hepatitis B Virus Large and Middle Envelope Proteins

**DOI:** 10.3390/v13040613

**Published:** 2021-04-02

**Authors:** Jing Zhang, Yongxiang Wang, Shuwen Fu, Quan Yuan, Qianru Wang, Ningshao Xia, Yumei Wen, Jisu Li, Shuping Tong

**Affiliations:** 1 Key Laboratory of Medical Molecular Virology, Department of Pathobiology, School of Basic Medical Sciences, Fudan University, Shanghai 200032, China; jing_zhang16@fudan.edu.cn (J.Z.); yxwang712@yahoo.com (Y.W.); fsw_1992@163.com (S.F.); 17211010049@fudan.edu.cn (Q.W.); ymwen@shmu.edu.cn (Y.W.); 2 State Key Laboratory of Molecular Vaccinology and Molecular Diagnostics, School of Public Health, Xiamen University, Xiamen 361102, China; yuanquan@xmu.edu.cn (Q.Y.); nsxia@xmu.edu.cn (N.X.); 3 Liver Research Center, Rhode Island Hospital, The Warren Alpert School of Medicine, Brown University, Providence, RI 02903, USA; ji_su_li_md@brown.edu

**Keywords:** hepatitis B virus, hepadnaviridae, hepatitis B surface antigen, envelope proteins, core protein, virions, subviral particles, secretion, virus evolution

## Abstract

Hepatitis B virus (HBV) expresses co-terminal large (L), middle (M), and small (S) envelope proteins. S protein drives virion and subviral particle secretion, whereas L protein inhibits subviral particle secretion but coordinates virion morphogenesis. We previously found that preventing S protein expression from a subgenomic construct eliminated M protein. The present study further examined impact of S protein on L and M proteins. Mutations were introduced to subgenomic construct of genotype A or 1.1 mer replication construct of genotype A or D, and viral proteins were analyzed from transfected Huh7 cells. Mutating S gene ATG to prevent expression of full-length S protein eliminated M protein, reduced intracellular level of L protein despite its blocked secretion, and generated a truncated S protein through translation initiation from a downstream ATG. Truncated S protein was secretion deficient and could inhibit secretion of L, M, S proteins from wild-type constructs. Providing full-length S protein in trans rescued L protein secretion and increased its intracellular level from mutants of lost S gene ATG. Lost core protein expression reduced all the three envelope proteins. In conclusion, full-length S protein could sustain intracellular and extracellular L and M proteins, while truncated S protein could block subviral particle secretion.

## 1. Introduction

Hepatitis B virus (HBV) is an enveloped DNA virus with a small circular genome of 3.2 kb [1,2]. The 1.2-kb coding sequence of the envelope gene is further divided into the preS1, preS2, and S regions, each starting with an ATG codon and having a coding capacity for 108 or 119 amino acids (aa), 55aa, and 226aa, respectively. Alternative translation initiation from these in-frame ATGs generates large (L), middle, and small (S) envelope proteins, with the preS1 domain present in just L protein while the preS2 domain present in both L and M proteins. Due to a facultative N-linked glycosylation site in the S domain, each envelope protein is present in two size forms: gp42 and p39 (L), gp36 and gp33 (M), and gp27 and p24 (S). L protein is translated from the 2.4-kb subgenomic RNA, while M and S proteins are products of the shorter (2.1-kb) RNA, the most abundant HBV transcript. Transcription of the 2.4-kb and 2.1-kb RNAs is driven by the SPI and SPII promoters, respectively, with the SPII promoter located in the preS region. The S protein is expressed at much higher level than L and M proteins and is the most abundant envelope protein on the 42-nm virions. Moreover, S and M proteins are also secreted without internal core particles as the 22-nm subviral particles (SVPs), which exceed virions by up to 100,000-fold [3,4,5]. Collectively the three envelope proteins on SVPs and virions are detected by anti-S antibody as hepatitis B surface antigen (HBsAg), which provides a sensitive serological marker of on-going HBV infection.

HBV genome replication initiates with the assembly of core protein into core particles, packaging pregenomic RNA (pgRNA). pgRNA is converted by the co-packaged DNA polymerase (P protein or pol) into partially double stranded DNA. L protein initiates virion morphogenesis by interacting with core particles through its matrix domain [6,7]. L protein also retains S protein for its incorporation into virions. The S protein drives the secretion of both virions and SVPs. In fact, S protein expressed alone is sufficient for SVP assembly and release, whereas L protein is not secreted without co-expression of S protein. L protein inhibits SVP secretion according to the L/S protein ratio while efficient virion secretion requires a proper (not too high or too low) L/S ratio [8,9,10,11]. The M protein is dispensable for the secretion of either virions or SVPs [12,13,14,15], and its exact function in HBV life cycle remains poorly defined.

**Figure 1:**
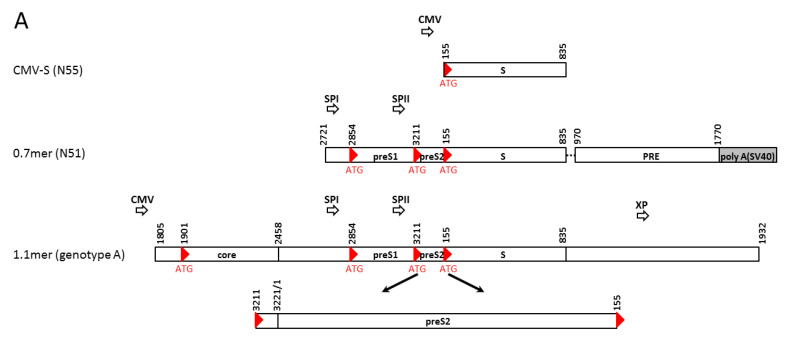

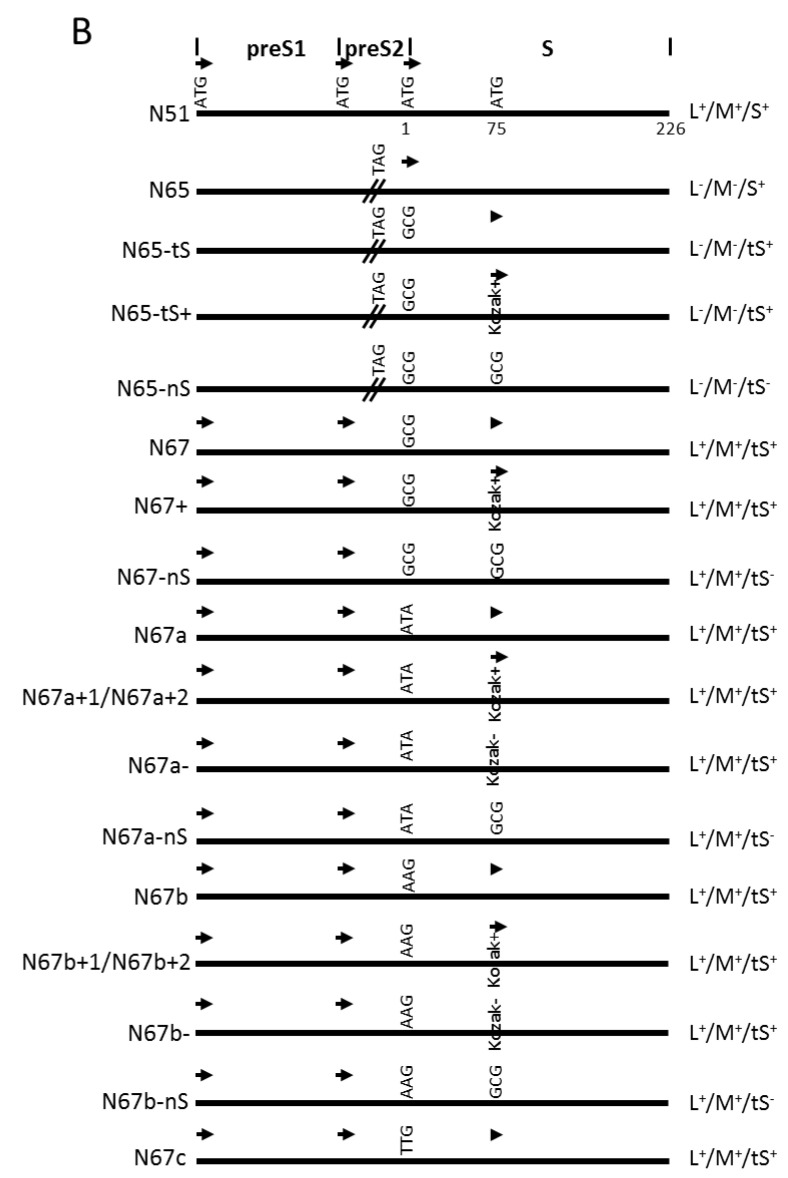
Schematic representation of the DNA constructs used in the present study and summary of their L, M, S (or tS) expression profiles. (**A**) Comparison among CMV-S construct, 0.7 mer subgenomic construct, and 1.1 mer replication construct of genotype A. The red triangles indicate translation initiation codons of the core gene, preS1, preS2, and S regions, respectively. Open arrowheads indicate the CMV, SPI, SPII, and X promoters. PRE: Posttranscriptional regulatory element. (**B**) The parental 0.7 mer L/M/S construct (N51) and site-directed mutants to alter L/M or S protein expression. Only the coding sequence of the envelope gene is shown. Arrowheads indicate sites of translation initiation. tS: truncated S protein through translation initiation from codon 75. For some constructs its Kozak sequence was optimized (+) or further weakened (−) to alter its expression level (as indicated by size of arrowhead). nS: no expression of full-length or truncated S protein through further mutation of codon 75.

A subgenomic (0.7 mer) construct of the HBV genome inserted to a cloning vector could express all the three envelope proteins under endogenous SPI and SPII promoters (Figure 1A). We previously found that preventing S protein expression from such a 0.7 mer construct nearly abolished M protein [12]. In the present study, we examined the impact of S protein co-expression on intracellular and extracellular levels of L protein, both in the context of 0.7 mer construct and full-length HBV genome (1.1 mer replication construct). We also began to test the impact of lost core protein expression on intracellular and extracellular levels of the three envelope proteins.

## 2. Materials and Methods

### 2.1. Subgenomic Expression Constructs for HBV Envelope Proteins and Site-Directed Mutants

Construct N55 had the S gene (nt155-835) of HBV6.2 of genotype A cloned to pcDNA3.1/Zeo(-) vector (Invitrogen, Carlsbad, CA, USA) to allow S protein expression under the CMV promoter (Figure 1A). The 0.7 mer construct had nt2721-3221/1-835 of a genotype A clone containing the entire envelope gene in addition to both SPI and SPII promoters inserted to the SacI—HindIII sites of pBluescript SK(-) vector (Stratagene, La Jolla, CA, USA). This was followed by HBV posttranscriptional regulatory element (PRE: nt970-1770) and a 300-bp SV40 polyadenylation signal (via HindIII—XhoI sites) (Figure 1A) [16]. The wild-type (WT) construct, called N51, could efficiently express all the three envelope proteins [12]. Expression of full-length S protein from the 0.7 mer construct was prevented by mutating the S gene start codon into GCG, ATA, AAG, or TTG (N67, N67a, N67b, N67c; Figure 1B; Table 1), while expression of intact L and M proteins was abolished by mutating preS2 codon 43 from TCG to TAG (N65). All the mutations were introduced by overlap extension PCR followed by replacement of the SacI—HindIII fragment. Subsequent experiments revealed that constructs with mutated S gene ATG could express a shortened S protein by translation initiation from the second in-frame ATG codon (codon 75). That was abolished by mutating codon 75 from ATG to GCG (N65-nS, N67-nS, N67a-nS, N67b-nS; Figure 1B). Alternatively, expression of the truncated S protein was enhanced by mutating its Kozak sequence from CTGGATGTGTCTG to CGGGATGGGTCTG (N67a+1, N67b+1) or CAGGATGGGTCTG (N67a+2, N67b+2, N67+, N65-tS+), and diminished by its mutation to TTTTATGTGTCTT (N67a-, N67b-). Many mutations in the S region caused amino acid substitutions in L and M proteins, and sometimes in S protein if it was expressed (Table 1).

### 2.2. 1.1 mer Constructs and Mutation to Prevent the Expression of S, L, or Core Protein

The 1.1 mer construct was generated by inserting nt1805-3221/1-1932 of a genotype A clone or nt1805-3182/1-1932 of two genotype D clones into the SacI—HindIII sites of pcDNA3.1 zeo(-) vector (Figure 1A). The genotype A clone (geno5.4; GenBank accession number KX827293) and one genotype D clone (geno1.2; KX827290) were from our previous collection [17], while the other genotype D clone was the first sequenced HBV genome and infectious in cell culture (V01460) [18,19]. The core^-^ mutant had C48* nonsense mutation (C2044A in geno5.4 and T2044A in geno1.2 at the nucleotide level) (Table 2). The L^-^ mutant had W52* (G3008A) nonsense mutation for geno5.4 but K38* (A2959T) nonsense mutation for geno1.2, with the latter accompanied by Q218L substitution in P protein. The S^-^ mutant of all the three clones had the S gene start codon mutated to ACG. Two preS2 mutations were introduced to the infectious genotype D clone [18,19]. They target preS2 residues 13–16 (QDPR), a neutralizing epitope [20]. The four residues were deleted in del13–16 and mutated to alanine in the AAAA mutant (see Table 2 for changes at the nucleotide level and impact on P protein).

### 2.3. Transient Transfection and Western Blot Analysis

The transfection experiments and Western blot analysis are well established [12,16,17,19,21,22,23,24]. The human hepatoma cell line Huh7 was cultured in Dulbecco’s Modified Eagle’s Medium (Gibco, Gaithersburg, MD, USA) supplemented with 10% fetal bovine serum (Gibco), 100 U/mL penicillin and 100 μg/mL streptomycin (Gibco). Cells were seeded into 6-well plates and transfected 18–24 h later, when the cell density reached about 80%. Plasmid DNA (2 μg/well) was transfected using Lipofectamine™ 3000 Transfection Reagent (Invitrogen). Cells and culture supernatant were harvested 72 h later. Cells were lysed in 120 μL lysis buffer consisting of 10 mM HEPES, pH 7.5; 100 mM NaCl; 1 mM EDTA; 1% NP40 and cOmplete™ protease inhibitor cocktail (Roche). Proteins from 1/15th cell lysate or precipitated from 100 μL of culture supernatant by 10% polyethylene glycol (PEG) were separated by SDS-polyacrylamide gel electrophoresis. Following transfer, the Western blots were incubated at 4 °C overnight with mouse monoclonal anti-preS1 (7H11; 1:4000), rabbit polyclonal anti-preS2 (GenScript, Shanghai, China; 1:2000), anti-HBs antibody (Novus, Centennial, CO, USA; 1:3000) or anti-HBc antibody (2A7; 1:10,000) diluted in 5% milk-TBST. The blots were washed for 30 min in TBST and incubated for 1 h with a 1:8000 dilution of goat anti-rabbit or anti-mouse antibody conjugated with horse radish peroxidase (HRP). The blots were washed for 30 min and signals were detected by Western Lightning Plus-ECL, Enhanced Chemiluminescence Substrate (Perkin-Elmer, Shelton, CT, USA). As a loading control, glyceraldehyde-3-phosphate dehydrogenase (GAPDH) was detected by a mouse monoclonal antibody (Proteintech, 1:5000 dilution) followed by anti-mouse-HRP (1:10,000). Multi Gauge V2.2 software was used to measure the grey values of signals on the blots.

### 2.4. ELISA Measurement of HBsAg and preS1 Antigen

HBsAg and preS1 antigens in culture supernatant and cell lysate were measured by enzyme linked immunosorbent assay (ELISA) using commercial kits (KHB, Shanghai, China) according to the manufacturer’s direction. Different dilutions were used for samples with high vs. low HBsAg and preS1 antigen titers to avoid signal saturation.

### 2.5. Southern Blot Analysis of Replicative DNA and Virion DNA

A total of 2 μg plasmid DNA was transfected into Huh7 cells seeded in 6-well plates. Cells and culture supernatant were harvested four days later. Details of HBV DNA analysis have been described [12,19,21,22,24]. Cells were lysed in 120 μL of lysis buffer containing 10 mM HEPES (pH7.5), 100 mM NaCl, 1 mM EDTA, and 1% NP40. A 1/3rd of the lysate was treated with 7.5U of mung bean nuclease (New England Biolabs, Ipswich, MA, USA) and 5U of DNase I (Roche) at 37 °C for 30 min in the presence of 10 mM CaCl_2_ and 12 mM MgCl_2_. Core particles were precipitated overnight by adding 38 μL of PEG solution (1.2M NaCl, 60 mM EDTA, 30% sucrose, 26% PEG8000), resuspended in 50 μL of 10 mM Tris (pH7.5)/8 mM CaCl_2_/6 mM MgCl_2_, and further treated with 3U of mung bean nuclease and 2U of DNase I at 37 °C for 30 min. Core particles were disrupted by addition of 150 μL of proteinase K digestion buffer (25 mM Tris pH 7.5, 10 mM EDTA, 100 mM NaCl, 0.5% SDS), and digested overnight at 55 °C with 0.5 mg/mL of proteinase K (Sangon Biotech, Shanghai, China). DNA was extracted with Tris-saturated phenol (Sangon Biotech), and precipitated with ethanol. Purified DNA was dissolved in Tris buffer and separated in 1.3% agarose gel. Following overnight transfer to a nylon membrane (Roche), the blot was hybridized with α-^32^P-labeled probe of genotype D (purified 3.2-kb HBV DNA devoid of vector sequence). The signals on the phosphor screen were scanned by Typhoon FLA 9500 software. Virions were immunoprecipitated from 1.5 mL of culture supernatant by overnight incubation at 4 °C with rabbit anti-preS1 antibody (Genscript) and rabbit anti-S antibody (Novus) preconjugated to protein G-Sepharose beads. The precipitate was washed with PBS buffer twice and resuspended in 50 μL of 10 mM Tris (pH 7.5)/8 mM CaCl_2_/6 mM MgCl_2_. Samples were treated with 3U of mung bean nuclease and 2U of DNase I at 37 °C for 30 min. This was followed by addition of 150 μL of the proteinase K buffer, and digestion with 0.5 mg/mL of proteinase K at 55 °C overnight. DNA was extracted with Tris-saturated phenol and precipitated with ethanol using 1 μL salmon sperm DNA and 1 μL glycogen (Roche) as carriers. Purified virion DNA was subject to Southern blot analysis just as for intracellular replicative DNA.

### 2.6. Statistical Analysis

All experiments were repeated for three times, and the data were presented as mean with standard deviation (SD). Statistical analysis was performed by GraphPad Prism 6 software using a Student’s *t*-test. * *p* < 0.05 is considered as statistically significant.

## 3. Results

### 3.1. Mutating the S Gene ATG Codon from a Subgenomic L/M/S Construct of a Genotype A Clone Reduced Intracellular Level of L Protein Despite Its Blocked Secretion

**Figure 2:**
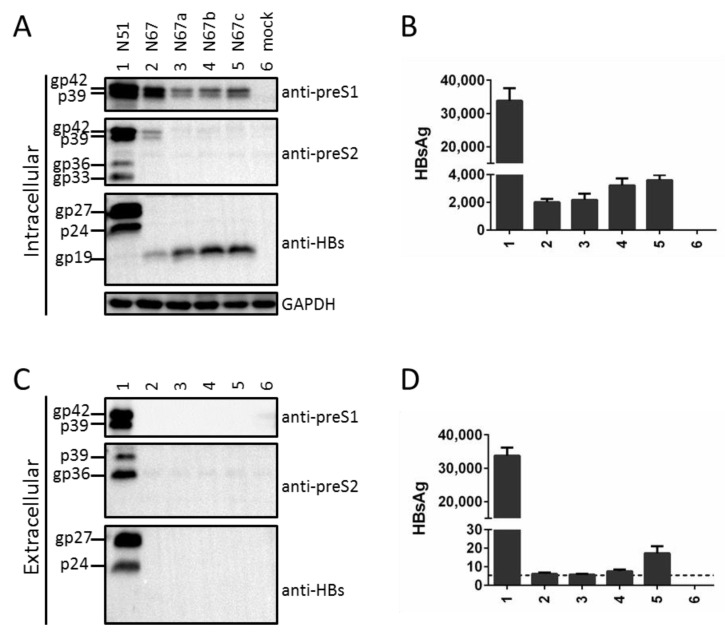
Preventing S protein expression from 0.7 mer construct by mutating the translation initiation codon not only blocked L protein secretion but also markedly reduced its intracellular level. N67, N67a, N67b, and N67c were derived from N51 by mutating the S gene ATG codon into different codons to prevent expression of full-length S protein. (**A**) Western blot analysis of intracellular L, M, and S proteins with GAPDH serving as a loading control. gp19 is N-terminally truncated S protein. (**B**) ELISA quantification of intracellular HBsAg. (**C**) Western blot analysis of secreted L, M, and S proteins following PEG precipitation. (**D**) ELISA quantification of secreted HBsAg.

We previously generated N51 (L^+^/M^+^/S^+^), a 0.7 mer construct of a genotype A clone capable of efficient expression of the three envelope proteins under endogenous SPI and SPII promoters [12]. Mutating its S gene ATG codon into GCG (N67) eliminated both glycosylated and nonglycosylated forms of S protein (gp27 and p24), from both lysate and culture supernatant of transiently transfected Huh7 cells (Figure 2A,C, third panels, compare lanes 1 and 2). Surprisingly, intracellular M protein (gp36 and gp33) also became undetectable while the L protein (gp42 and p39) was much reduced (Figure 2A, top two panels, lanes 1 and 2). This was striking considering that lost S protein expression prevented L protein secretion (Figure 2C, top panel), which should increase its intracellular level. The ATG-to-GCG change in N67 caused M175A substitution in L protein (Table 1). In case this substitution reduced L protein level, we further mutated the S gene ATG into ATA, AAG, or TTG (N67a, N67b, N67c). However, similar to N67, all the three mutants lost S (gp27/p24) and M (gp36/gp33) proteins in cell lysate and HBsAg in culture supernatant (Figure 2A,D, lanes 3–5). Despite lost L protein in culture supernatant (Figure 2C, top panel), the three mutants showed even lower intracellular level of L protein than N67 (Figure 2A, top panel, compare lanes 3–5 with lane 2). Therefore, preventing expression of the full-length S protein by mutating the translation initiation codon dramatically reduced total (intracellular + extracellular) amount of L protein.

### 3.2. Mutating the S Gene ATG Codon Led to Translation Initiation from the Next In-Frame ATG to Generate an N-Terminally Truncated S Protein

**Figure 3:**
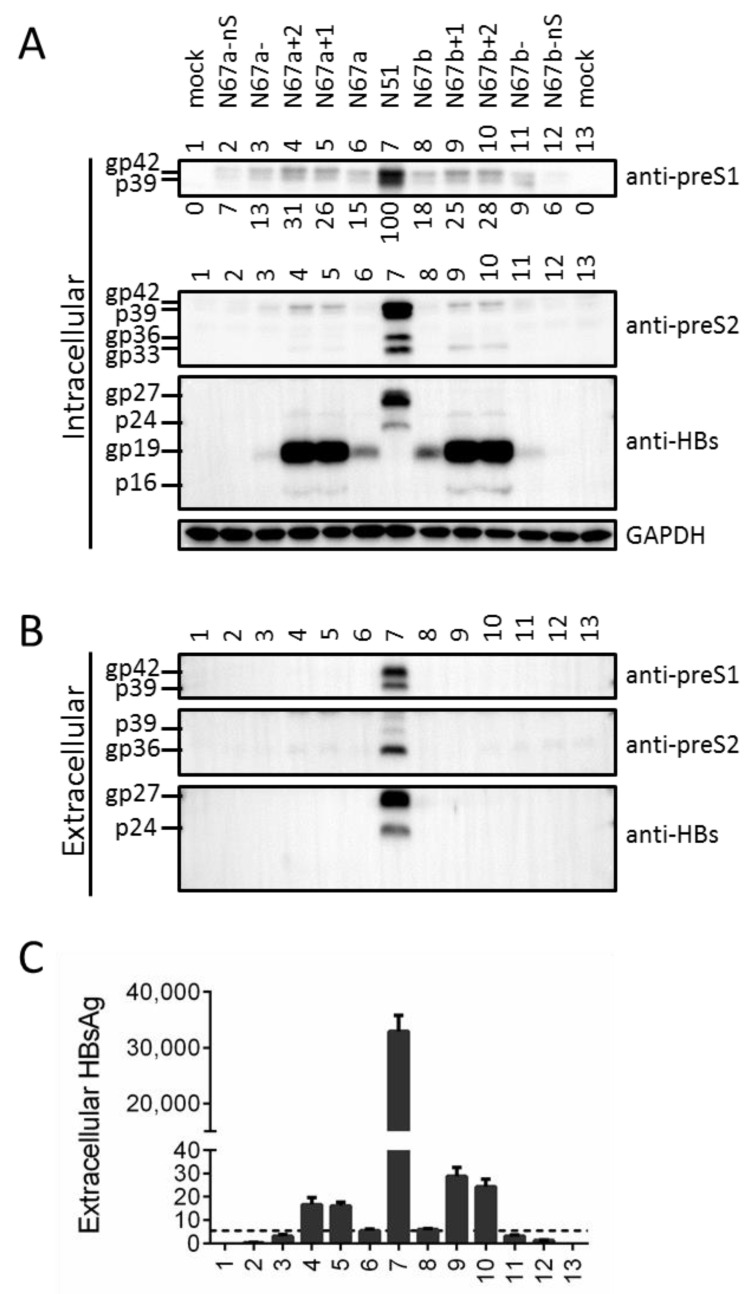
Establishment of gp19 as N-terminally truncated S protein and confirmation of its ability to partially sustain intracellular level of L protein in cis. pg19 expression from N67a and N67b was increased or decreased by mutating the Kozak sequence (N67a+1, N67a+2, N67a-, N67b+1, N67b+2, N67b-) or prevented by mutating codon 75 of the S gene from ATG into GCG (N67a-nS, N67b-nS). (**A**,**B**) Intracellular (**A**) and extracellular (**B**) L, M, and S proteins, with GAPDH serving as intracellular control. (**C**) Extracellular HBsAg. The numbers below the anti-preS1 blot in panel A represent the amount of L protein for each construct relative to N51 (set at 100) in three independent transfection experiments.

**Figure 4:**
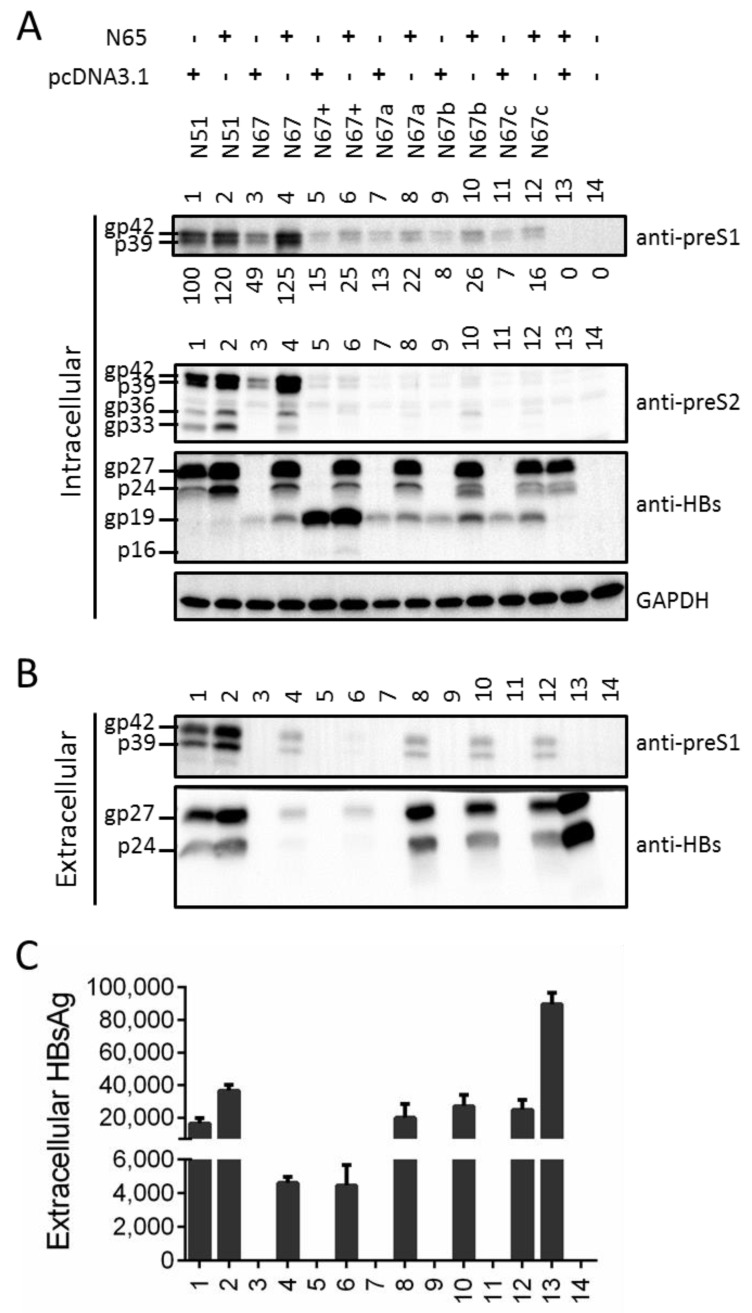
Providing full-length S protein in trans rescued L protein secretion and moderately increased its intracellular level in the context of 0.7 mer construct. The parental construct (N51) and its 5 mutants incapable of expressing full-length S protein (N67, N67+, N67a, N67b, N67c) were co-transfected with the S protein expression construct N65 or pcDNA3.1 at 1 μg:1 μg ratio. (**A**,**B**) Intracellular (**A**) and extracellular (**B**) L and S proteins, with GAPDH serving as intracellular control. (**C**) Extracellular HBsAg. Note that providing full-length S protein in trans failed to rescue gp19 secretion. The numbers below the anti-preS1 blot in panel A represent the amount of L protein for each lane relative to lane 1 (set at 100) in three independent transfection experiments.

Low amount of HBsAg could be detected from lysate of Huh7 cells transfected with the four mutants of S gene ATG (Figure 2B, lanes 2–5), and Western blot with the anti-S antibody revealed a protein band of about 19 kd (Figure 2A, third panel). In this regard the next in-frame ATG codon in the S gene is codon 75, and its translational initiation should generate a truncated S (tS) protein of 152aa (about 19 kd if modified by N-linked glycosylation). Optimizing the Kozak sequence of that ATG codon for N67a (N67a+1, N67a+2) or N67b (N67b+1, N67b+2) dramatically enhanced the 19-kd band in cell lysate and created a new band of 16 kd (Figure 3A, third panel, compare among lanes 4–6; and among lanes 8–10), and the same was true for N67+ relative to N67 (Figure 4A, third panel, lanes 3 and 5). gp19 was reduced by weakening the Kozak sequence (N67a-, N67b-), and abolished by mutating codon 75 to GCG (N67a-ns, N67b-ns) (Figure 3A, third panel, lanes 3, 11; and lanes 2, 12). These results demonstrated that gp19 was a truncated S protein through translation initiation from the second in-frame ATG codon in the S gene.

### 3.3. Truncated S Protein Was Secretion-Deficient but Capable of Partially Sustaining Intracellular Level of L Protein When Provided in Cis

No gp19 was detected from culture supernatant, even for cells transfected with N67a+1, N67a+2, N67b+1, or N67b+2 (Figure 3B, bottom panel, lanes 4, 5, 9, 10), suggesting that the truncated S protein was not secreted in contrast to the full-length protein. Consequently for such mutants, little HBsAg was detectable from culture supernatant (Figure 3C) and no L protein was secreted (Figure 3B, top panel, lanes 4, 5, 9, 10). Intracellular L protein was modestly increased for these four mutants but reduced for mutants with lost gp19 expression (N67a-ns, N67b-ns) (Figure 3A, top panel, lanes 4, 5, 9, 10 vs. lanes 2, 12). This was confirmed by three independent transfection experiments followed by densitometric analysis of signals on the Western blots (numbers shown below top panel in Figure 3A). Much increased gp19/p16 expression also led to the reappearance of M protein for N67b+1 and N67b+2 (Figure 3A, second panel, lanes 9, 10). These findings suggest that truncated S protein could sustain intracellular level of L protein, but not as efficiently as the full-length S protein even when expressed at higher level.

### 3.4. Providing Full-Length S Protein in Trans Rescued L Protein Secretion from ATG Codon Mutants and Moderately Increased Its Intracellular Level

The S gene codes for the S domain of L and M proteins, and the point mutations in N67, N67a, N67b, and N67c to abolish expression of full-length S protein also caused single (but different) substitutions in L protein: M175A, M175I, M175K, and M175L (Table 1). To validate a role of full-length S protein in sustaining the intracellular level of mutant L proteins, we co-transfected N67, N67a, N67b, N67c, and N67+ at 1:1 ratio with N65 (L^-^/M^-^/S^+^) to provide full-length S protein in trans. Alternatively they were co-transfected with empty vector (pcDNA3.1) to serve as a negative control. N65 was derived from N51 (L^+^/M^+^/S^+^) by a nonsense mutation of preS2 codon 43 (S43*). N65 was also co-transfected with pcDNA3.1 or N51. Its co-transfection with N51 moderately increased intracellular levels of L and M proteins (Figure 4A, top two panels, compare lanes 1 and 2) as well as extracellular L protein (Figure 4B, top panel). Among the mutants incapable of expressing full-length S protein, N67 showed most increase in the total amount of intracellular L protein although the fold increase was also striking for N67b (Figure 4A, top panel, compare lanes 3 and 4; lanes 9 and 10. The numbers below the blot were based on densitometric analysis of Western blot images from three transfection experiments). Rescue of L protein secretion was least efficient for N67+ (Figure 4B, top panel, lanes 5, 6). Co-transfection with N65 also increased intracellular gp19 (Figure 4A, third panel, compare between odd and even numbered lanes), suggesting its stabilization by the full-length S protein. Consistent with the ability of L protein to inhibit S protein secretion [8,9,10,11], highest extracellular gp27/p24 and HBsAg were achieved by N65 co-transfection with empty vector (Figure 4B,C, lane 13). Lowest extracellular gp27/p24 and HBsAg were produced by N65 co-transfection with N67 or N67+ (lanes 4 and 6). N65 co-transfection with N67+ led to less extracellular L protein than its co-transfection with N67 (Figure 4B, top panel, lanes 4 and 6), although the intracellular level was also lower (Figure 4A, top panel).

### 3.5. Providing Truncated S Protein in Trans Failed to Rescue L Protein Secretion from ATG Codon Mutants but Could Increase Intracellular L Protein

**Figure 5:**
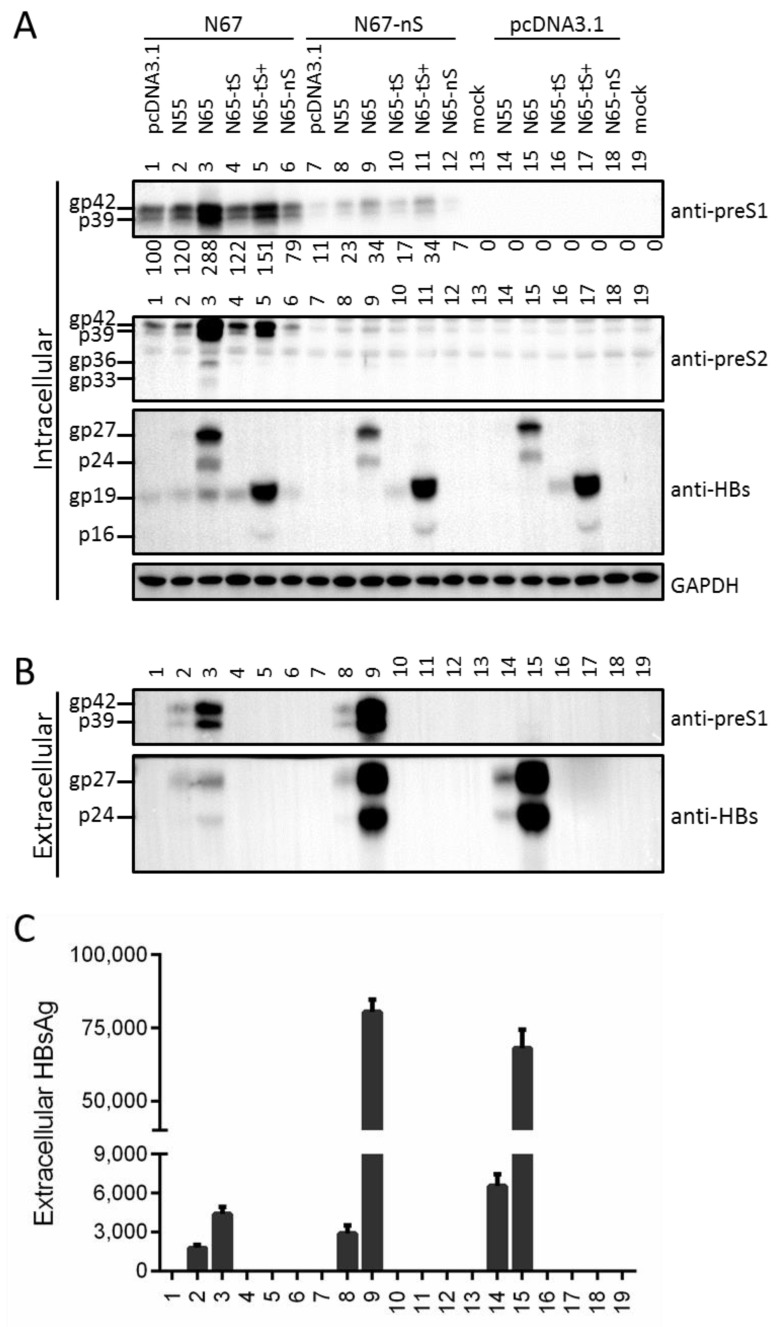
Providing truncated S protein in trans failed to rescue L protein secretion but somewhat increased its intracellular level in the context of 0.7 mer construct. N67 (incapable of expressing full-length S protein), N67-nS (incapable of expressing full-length or truncated S protein), or pcDNA3.1 was co-transfected with expression constructs for full-length S protein (N55, N65) or truncated S protein (N65-tS, N65-tS+), or control plasmids (pcDNA3.1, N65-nS) at 1 μg/1 μg ratio. (**A**,**B**) Intracellular (**A**) and extracellular (**B**) L and S proteins, with GAPDH serving as intracellular control. (**C**) Extracellular HBsAg. The numbers below anti-preS1 blot in panel A represent the amount of L protein in each lane relative to lane 1 (set at 100) in three independent experiments.

Although data presented in Figure 3A suggested ability of truncated S protein to sustain intracellular level of L protein, mutations to increase, reduce, or to abolish its expression were accompanied by substitutions in L protein (Table 1) which might alter L protein stability. To further validate the ability of truncated S protein to sustain intracellular L protein and to compare truncated S protein with full-length S protein, we generated an expression construct for truncated S protein (N65-tS) by mutating the S gene ATG of N65 into GCG (Figure 1B & Table 1). Furthermore, its expression was enhanced by optimizing the Kozak sequence (N65-tS+) and abolished by mutating codon 75 into GCG (N65-nS) (Figure 5A, third panel, compare lanes 16, 17, 18). Inserting the S gene behind the CMV promoter generated a construct with much reduced S protein expression than N65 (N55; Figure 5B, bottom panel, compare lanes 14 and 15). These constructs were co-transfected with N67 or N67-ns (both incapable of expressing full-length S protein, with N67-nS also incapable of expressing truncated S protein), or pcDNA3.1 (as a control). Although N55 and N65 produced less intracellular gp27/p24 than gp19/p16 produced by N65-tS and N65-tS+, respectively (Figure 5A, third panel, compare lanes 14, 15 with lanes 16, 17), they were not less efficient at increasing intracellular level of L protein from N67 (Figure 5A, top panel, compare lanes 2, 3 with lanes 4, 5) or N67-nS (compare lanes 8, 9 with lanes 10, 11). In fact N65 increased intracellular L and M proteins for N67 better than N65-tS+. Comparison between N55 and N65, or between N65-tS and N65-tS+, suggested dose-dependent effect for both full-length and truncated S protein. No gp19 or p16 was secreted even following N65-tS+ co-transfection with pcDNA3.1 (Figure 5B, lower panel, lane 17). N65 rescued L protein secretion much more efficiently for N67-nS than for N67 (Figure 5B, top panel, compare lanes 9 and 3). In this regard L protein from N67-nS failed to inhibit S protein secretion from N65, with HBsAg value similar to N65 co-transfection with pcDNA3.1 (Figure 5B,C, lanes 9 and 15).

### 3.6. Truncated S Protein Had Dominant Negative Effect on HBsAg Secretion from Wild-Type 0.7 mer L^+^/M^+^/S^+^ Construct of Genotype A or 1.1 mer Construct of Genotype A or D

**Figure 6:**
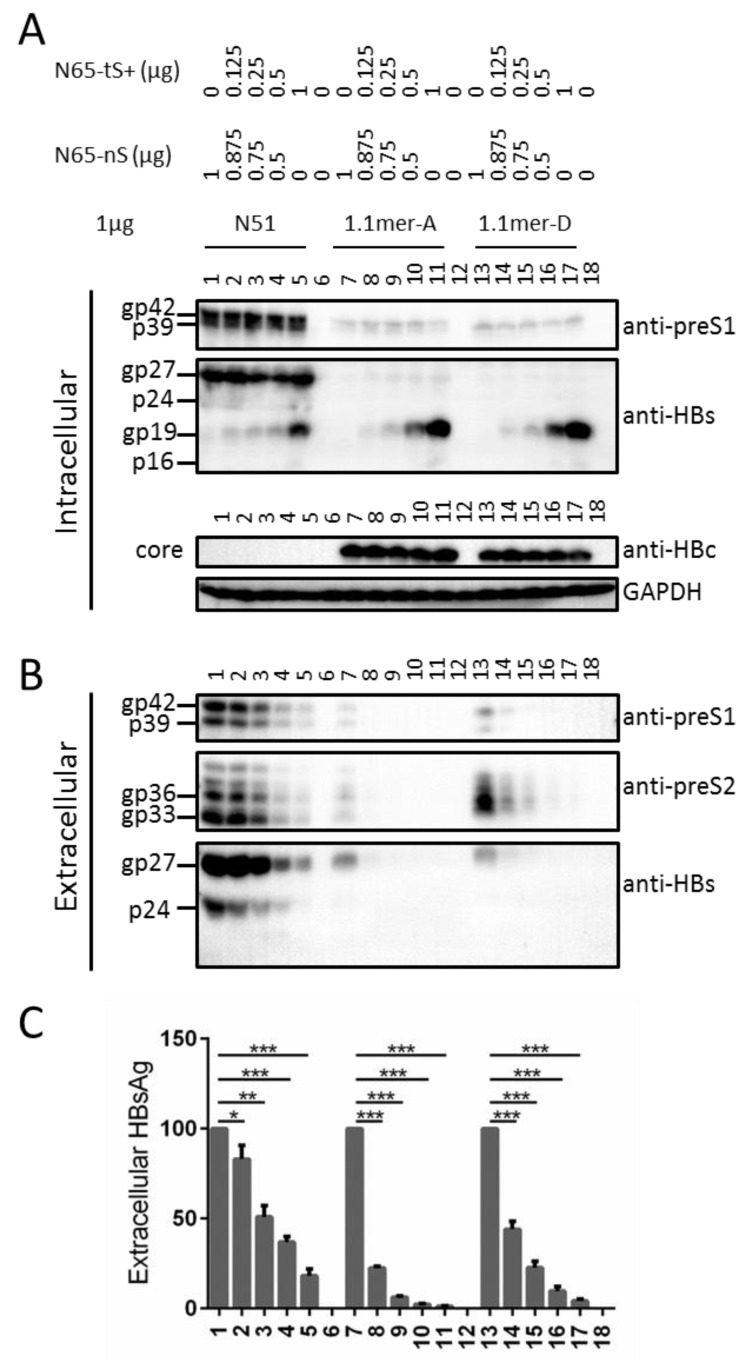
Providing truncated S protein in trans could inhibit L, M, and full-length S protein secretion from wild-type 0.7 mer L/M/S construct and 1.1 mer replication construct. Fixed amount (1 μg) of 0.7 mer construct of genotype A (N51) or 1.1 mer construct of genotype A (geno5.4) or D (geno1.2) was co-transfected with 0–1 μg of N65-tS+ overexpressing gp19/p16, with the total amount of co-transfected DNA kept at 1 μg using N65-nS. (**A**) Intracellular L, S, and core proteins, with GAPDH serving as a loading control. (**B**) Extracellular L, M, and S proteins. (**C**) Extracellular HBsAg, with values for N51, 1.1 mer-A, and 1.1 mer-D without co-transfection with N65-tS+ set at 100. * *p* < 0.05; ** *p* < 0.01; *** *p* < 0.001.

The fact that truncated S protein was deficient in secretion but could increase intracellular level of L protein raised possibility of its inhibition of L or S protein secretion from the wild-type HBV constructs. Towards this end fixed amount of the 0.7 mer construct N51 (L^+^/M^+^/S^+^) or 1.1 mer replication construct of genotype A (geno5.4) or genotype D (geno1.2) was co-transfected with increasing amount of N65-tS+ overexpressing gp19/p16, with the total amount of co-transfected plasmid DNA kept constant using N65-nS (incapable of expressing any envelope protein). Although the intracellular level of gp19/p16 increased according to the amount of N65-tS+ co-transfected, the intracellular level of L protein and full-length S protein did not show a clear dose-dependent increase (Figure 6A, top and second panels). For the 1.1 mer construct intracellular level of core protein was also unaffected (Figure 6A, third panel). However, extracellular L, M, and full-length S proteins showed a dose-dependent decrease for the 0.7 mer construct and 1.1 mer construct of genotype A or D (Figure 6B), and ELISA of extracellular HBsAg showed the same trend (Figure 6C). The reduction was less pronounced for the 0.7 mer construct possibly due to much higher basal level of S protein production and secretion (Figure 6A, second panel, Figure 6B, bottom panel: compare lanes 1, 7, 13). For the 1.1 mer construct, inhibition of HBsAg secretion was more dramatic for the genotype A clone (Figure 6C, compare lanes 7–11 with lanes 13–17).

### 3.7. Full-Length S Protein Sustained Intracellular Level of L Protein in the Context of a 1.1 mer Construct of Genotype D with Mutated S Gene ATG

**Figure 7:**
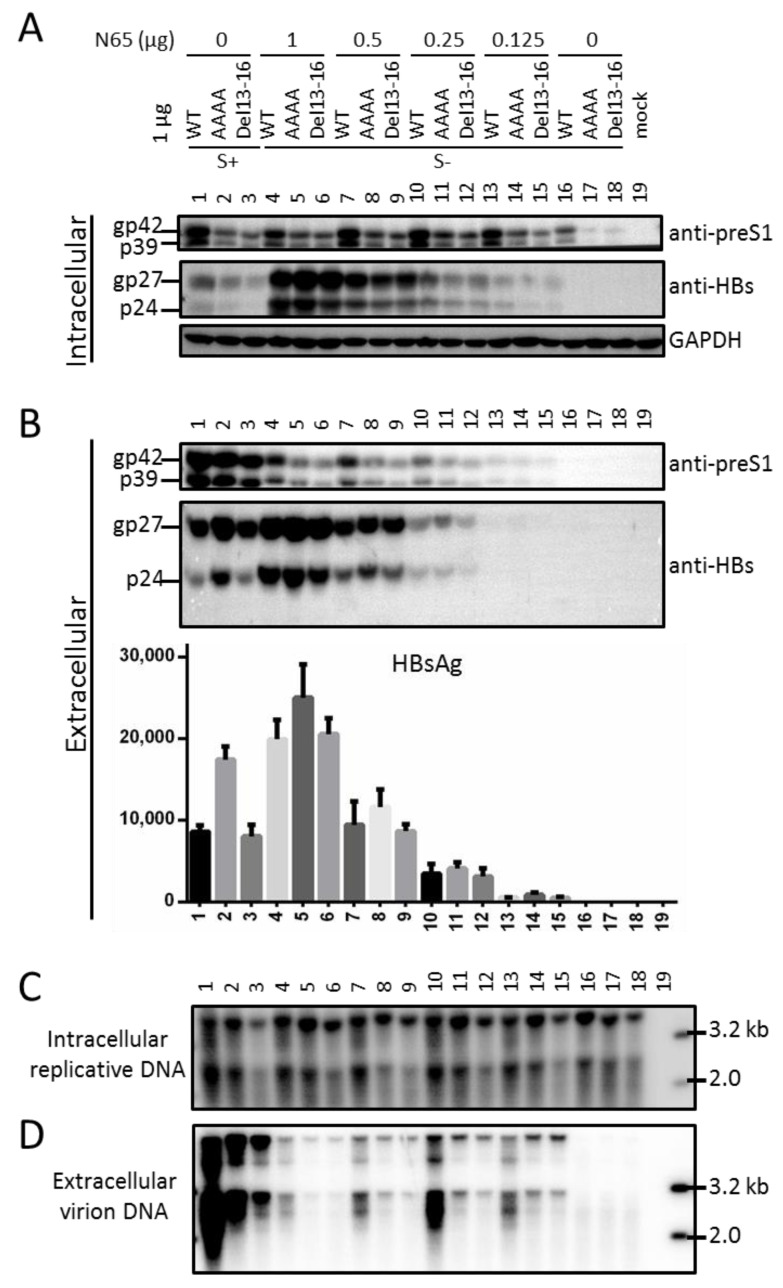
Providing full-length S protein in trans rescued L protein secretion and increased its intracellular level in the context of a 1.1 mer construct of genotype D incapable of gp27/p24 expression. Huh7 cells were transfected with 1 μg of a WT genotype D clone, its AAAA or del13–16 mutant (lanes 1–3), or corresponding S^-^ mutant with or without co-transfection with 0.125–1 μg of N65 as the source of full-length S protein (lanes 4–18). The total amount of DNA transfected was kept at 2 μg for all constructs by adding variable amount of pcDNA3.1 if necessary. (**A**) Intracellular L and S proteins, with GAPDH serving as a loading control. (**B**) Extracellular L, S proteins and HBsAg. (**C**) Intracellular replicative DNA. (**D**) Extracellular virion DNA.

So far, all the findings regarding the full-length S protein were based on the 0.7 mer L/M/S construct of a genotype A clone. Considering that L protein coordinates virion morphogenesis by interacting with both S and core proteins [3,4,5,6,7,25], it was necessary to validate a role of full-length S protein in sustaining the intracellular level of L protein in the context of genome replication and virion production, and in a different HBV genotype. A 1.1 mer construct was used, for which the strong CMV promoter drives efficient transcription of pgRNA, the genome precursor and mRNA for both core and P proteins. Besides the WT genotype D clone [18,19], its AAAA and del 13–16 mutants had preS2 residues 13–16 mutated to alanine and deleted, respectively. Both mutants had reduced intracellular levels of L protein than the WT construct, and the AAAA mutant showed increased HBsAg secretion (Figure 7A, top panel; Figure 7B, bottom panel, lanes 1–3). Preventing expression of full-length S protein by mutating the S gene ATG into ACG not only abolished L protein secretion, but also markedly reduced its intracellular level (Figure 7A,B, top panel, compare lanes 16–18 with lanes 1–3). Co-transfecting 1 μg of such S^-^ mutants with 0.25 or 0.5 μg of N65 was sufficient to markedly increase intracellular level of L protein (Figure 7A, top panel, compare lanes 7–9 and 10–12 with lanes 16–18). L protein became secreted, although not to the level of the parental WT, AAAA, or del13–16 construct (Figure 7B, top panel). Both intracellular gp27/p24 and extracellular HBsAg increased according to the amount of N65 co-transfected (Figure 7A, middle panel; Figure 7B, bottom panel). On the other hand, rescue of virion secretion was most effective when 0.25 μg of N65 was co-transfected, at least for the WT construct (Figure 7D, compare lane 10 with lanes 4, 7, 13).

### 3.8. Preventing Core Protein Expression from the 1.1 mer Construct Reduced Intracellular and Extracellular Levels of Envelope Proteins, for Both Genotype A and Genotype D

**Figure 8:**
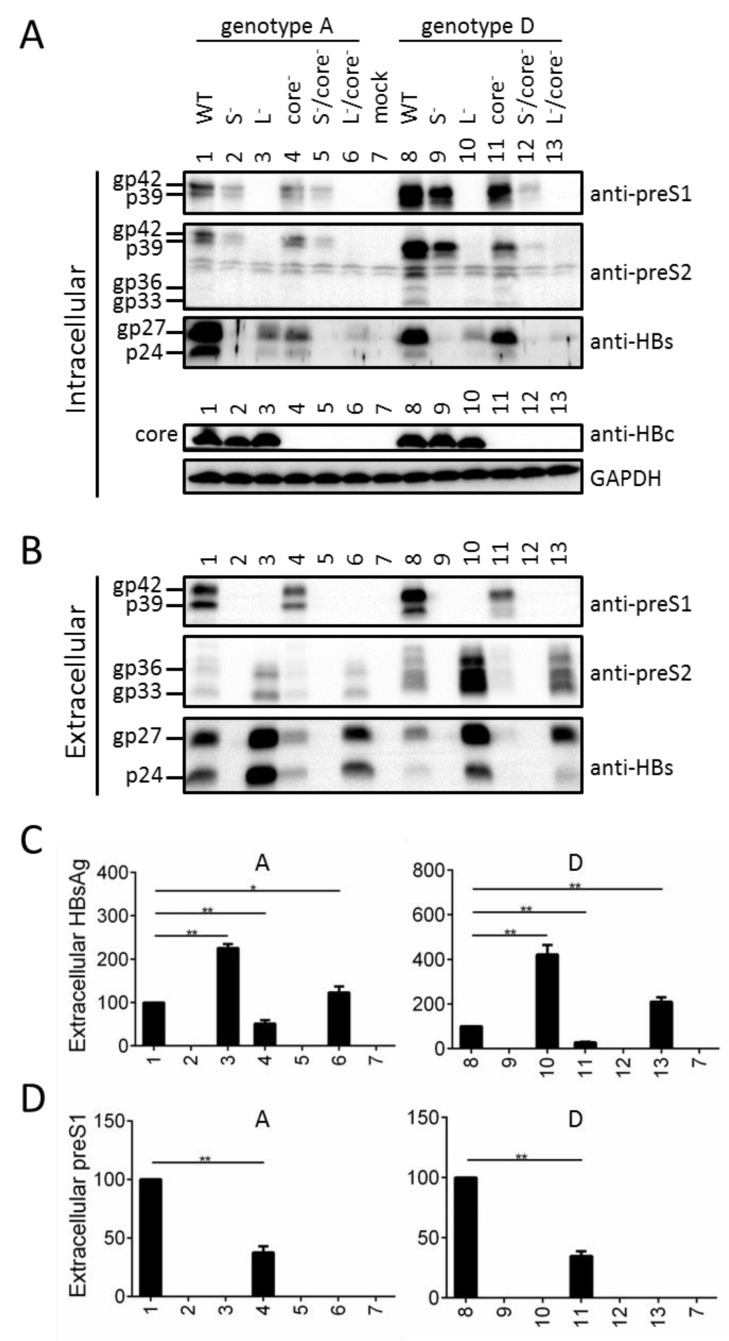
Loss of core protein expression reduced both the intracellular and extracellular levels of HBV envelope proteins in the context of a 1.1 mer construct of genotype A or D. The parental 1.1 mer construct (WT) of genotype A (geno5.4) or genotype D (geno1.2) and the 5 mutants deficient in expressing S, L, and/or core protein were transfected into Huh7 cells. (**A**) Intracellular L, M, S and core proteins, with GAPDH serving as loading control. (**B**) Extracellular L, M and S proteins. (**C**) Extracellular HBsAg with the WT value set at 100. (**D**) Extracellular preS1 antigen with the WT value set at 100. * *p* < 0.05; ** *p* < 0.01.

To test a possible role of core protein in sustaining the L protein, a core^-^ mutant was generated for the 1.1 mer construct of a genotype A clone (geno5.4) and a genotype D clone (geno1.2) by the C48* nonsense mutation (Table 2). Expression of full-length S protein was prevented by mutating the ATG codon into ACG, while L protein expression was abolished by the W52* nonsense mutation in genotype A but K38* nonsense mutation in genotype D (K38 in genotype D is analogous to K49 in genotype A due to N-terminal 11-aa deletion in this HBV genotype). The S^-^/core^-^ and L^-^/core^-^ double mutants were also generated. Preventing L protein expression promoted S protein secretion as evidenced by reduced intracellular level but increased extracellular level (Figure 8A,B, third panel, compare lanes 1 and 3; lanes 8 and 10; Figure 8C). According to anti-preS1 antibody, intracellular L protein was reduced in the S^-^ mutants but also in the core^-^ mutants (Figure 8A, top panel, compare among lanes 1, 2, 4; and among lanes 8, 9, 11). The level was further reduced in the S^-^/core^-^ double mutant, especially for genotype D (lanes 5 and 12). Blotting with the anti-preS2 antibody confirmed such a finding (Figure 8A, second panel), but also suggested a role of core protein in sustaining intracellular M protein for genotype D (intracellular M protein was undetectable for genotype A). Surprisingly, loss of core protein also reduced intracellular level of S protein (Figure 8A, third panel: compare lanes 1 and 4; 8 and 11; also compare lanes 3 and 6; 10 and 13). The reduction of intracellular L, M, and S proteins in the core^-^ mutants was not a consequence of their increased secretion, because their extracellular levels were reduced as well (Figure 8B). ELISA for HBsAg and the preS1 epitope in L protein confirmed findings according to Western blot analysis of extracellular S protein and L protein, respectively (compare Figure 8C with Figure 8B, bottom panel; compare Figure 8D with Figure 8B, top panel).

## 4. Discussion

We previously generated N51, a 0.7 mer construct of a genotype A clone for efficient expression of the three envelope proteins under endogenous HBV promoters [12]. The S^-^ mutant was generated by mutating its S gene ATG into GCG, as the double-nucleotide substitution should prevent translation initiation even from a non-ATG codon. Surprisingly, the mutant (N67) lost intracellular M protein and had much reduced L protein (Figure 2A, lane 2 vs. lane 1). Considering that lost S protein expression prevents the secretion of L protein (Figure 2C), this finding strongly suggested that co-expression of S protein is required to stabilize retained L and especially M proteins. Still, the ATG-to-GCG changes would introduce an M56A substitution in M protein and M175A substitution in L protein, which might be destabilizing. In the present study we generated three additional mutants, N67a, N67b, and N67c, with the S gene ATG mutated to ATA, AAG, or TTG (Figure 2). Furthermore, the ATG codon was mutated to ACG in another genotype A clone and two genotype D clones in the context of full-length HBV genome, which was silent in the overlapping P gene (Table 2; Figure 7 and Figure 8). The single nucleotide changes were sufficient to abolish expression of the full-length S protein, which was accompanied by blocked L protein secretion, lost intracellular M protein, and reduced intracellular L protein (Figure 2, lanes 3–5; Figure 7, lanes 16–18; Figure 8, lanes 2, 9). These findings further strengthen the notion that S protein co-expression is required to stabilize L and M proteins.

Nevertheless, data interpretation for these mutants was complicated by the detection of a new S protein of faster mobility (gp19 in Figure 2A), raising the possibility that lost or reduced intracellular M and L proteins was attributable to gp19 expression rather than lost gp27/p24 expression. Mammalian mRNA translation usually starts with the 5′ most ATG codon containing favorable Kozak sequence, and the second in-frame ATG in the S gene is codon 75 (M75 for S protein). Mutating that ATG eliminated gp19 (N67-nS, N67a-nS, N67b-nS), whereas optimizing its Kozak sequence dramatically increased gp19 and generated a 16-kd band (N67+, N67a+1, N67a+2, N67b+1, N67b+2) (Figure 3A and Figure 4A). Together, these findings unequivocally established gp19 as N-terminally truncated S protein through translation initiation from codon 75. Its identity as glycosylated form of p16 requires treatment of cell lysate with PNGase F, or culturing transfected cells in the presence of tunicamycin.

gp19 was deficient in secretion even when overexpressed (N67a+1, N67a+2, N67b+1, and N67b+2 in Figure 3B; N67+ in Figure 4B), which together with the absence of gp27/p24 could explain the lack of L protein secretion from all such mutants (Figure 3A and Figure 4A). gp19 remained non-secreted when co-expression of L and M proteins was prevented (N65-tS+ in Figure 5B, lane 17), and hence secretion deficiency is an intrinsic property of truncated S protein. For both N67a and N67b, intracellular L protein was increased by Kozak sequence mutations to up regulate gp19 (N67a+1, N67a+2, N67b+1, N67b+2), but reduced by mutated ATG codon to prevent its expression (N67a-nS, N67b-nS) (Figure 3A). In the co-transfection experiments, N65-tS+ was more efficient than N65-tS or N65-nS in increasing intracellular level of L protein for both N67 and N67-nS (Figure 5A). Thus N-terminally truncated S protein, when provided in cis or in trans at high amount, could sustain intracellular levels of L protein. Trans-complementation assay also confirmed ability of the full-length S protein to sustain intracellular level of L protein, whether in the context of the 0.7 mer construct (Figure 4 and Figure 5) or 1.1 mer construct (Figure 7). A possible mechanism whereby the S protein sustains L and M proteins is stabilization of the co-terminal L and M proteins through heterodimer formation via the shared S domain. Ability of truncated S protein to sustain intracellular levels of L and M proteins suggests that the N-terminal 74 residues of the S domain are not essential for heterodimer formation.

Since increased expression of gp19/p16 elevated rather than diminished intracellular level of L protein when the full-length S protein was absent (Figure 3A and Figure 5A), higher intracellular level of L protein displayed by N67 than N67a, N67b, and N67c (Figure 2A) was most likely attributed to different substitutions in L protein. Probably the M175A substitution present in N67 is less destabilizing than the M175I, M175K, or M175L substitution found in the other three mutants, or more efficient at retaining L protein. In the co-transfection experiment with N65, N67 inhibited full-length S protein/HBsAg secretion more potently than N67a, N67b, and N67c (Figure 4B,C), which should be attributed to difference in L protein rather than retention by truncated S protein (because N67 produced less gp19 than the other three constructs). Another meaningful comparison is between N67 and N67-nS. N67 displayed much more intracellular L protein than N67-nS, with or without co-transfection with an expression construct for the full-length or truncated S protein (Figure 5A, compare lanes 1–6 with lanes 7–12). Following co-transfection with N65, its extracellular L protein and full-length S protein/HBsAg were much lower than N67-nS (Figure 5B,C, lanes 3 and 9). In fact, the HBsAg titer of N67-nS co-transfection with N65 was similar to pcDNA3.1 co-transfection with N65, suggesting that the M249A substitution present in N67-nS but absent in N67, the parental construct, have rendered L protein no longer inhibitory of HBsAg secretion and more easily secreted with the help of full-length S protein. This low retention/high secretion property probably contributed to a low intracellular level of L protein for N67-nS than parental N67 with or without rescue by the full-length or truncated S protein.

We previously reported that preventing S protein expression from N51 via the ATG-to-GCG mutations of the S gene ATG codon abolished M protein [12]. In the present study we could reproduce the same effect by mutating the ATG into ATA, AAG, or TTG (Figure 2A), or by mutating the ATG codon to ACG in another genotype A clone and in a genotype D clone as 1.1 mer construct (Figure 8A,B). Providing full-length S protein in trans could rescue intracellular M protein for N67 and N67b (Figure 4A, lanes 4, 10; Figure 5A, lane 3), while increased expression of truncated S protein in N67b+1 and N67b+2 was accompanied by reappearance of M protein (Figure 3A, lanes 9 and 10). These findings strongly suggest a role of full-length S protein in promoting the secretion and stabilization of M protein, and ability of truncated S protein in stabilizing intracellular M protein. In this regard HBV is the prototype of hepadnaviridae, a family of hepatotropic DNA viruses infecting birds and mammals. Avian hepadnaviruses such as duck hepatitis B virus (DHBV) do not express M protein [26], and the later stage of chronic HBV infection often selects for point mutations of the preS2 ATG codon to ablate M protein expression [13,22,27,28,29,30,31]. Our current study also suggests a role of full-length or truncated S protein in stabilizing L protein. In this regard hepadnaviruses originated from nackednaviruses of fish, a non-enveloped virus lacking the preS region and having no coding capacity for S protein in the P gene [32]. Hepadnaviruses gained coding capacity for S protein in the RT/RNaseH coding sequence of P gene and had insertion of the preS region to code for the preS domain of L protein. Although transition from non-enveloped nackednavirus to enveloped hepadnaviruseses requires simultaneous expression of S and L protein (as neither one is sufficient for virion production), our findings raised possibility that S protein (and ability to secrete SVPs) might have evolved earlier than L protein.

The current study started to explore a possible role of core protein in sustaining the envelope proteins. We found that lost core protein expression via the C48* nonsense mutation diminished both intracellular and extracellular levels of L protein (Figure 8A,B, compare WT with core^-^ mutant; Figure 8A, compare S^-^ mutant with S^-^/core^-^ mutant). The S^-^/core^-^ mutant displayed even lower intracellular level of L protein than either S^-^ or core^-^ mutant (Figure 8A, compare lanes 2, 4, 5, and lanes 9, 11, 12), suggesting that S and core proteins sustain L protein through different mechanisms. Surprisingly, lost core protein expression also reduced intracellular and extracellular S and M proteins for genotype D, intracellular S protein and extracellular S and M proteins for genotype A (its intracellular M protein was barely detectable) (Figure 8A,B, top two panels, compare WT construct with core^-^ mutant, and L^-^ mutant with L^-^/core^-^ mutant). While provocative, these findings are preliminary. We cannot exclude the possibility that lost core protein expression somehow reduced 2.4-kb and 2.1-kb RNAs for envelope proteins. It remains to be determined whether similar phenotypes can be reproduced by ablating core protein expression through a different mutation (such as lost ATG codon). If the core protein can indeed stabilize the three envelope proteins, then co-transfection of the 1.1 mer core^-^ mutant with an expression construct for core protein should rescue intracellular and extracellular levels of L, M, and S proteins.

The present study demonstrated that deleting the N-terminal 74 residues from S protein could block its secretion. Providing such a truncated S protein in trans (N65-tS+) could dose-dependently inhibit HBsAg secretion from the WT construct, whether as 0.7 mer construct or 1.1 mer replication construct (Figure 6C). Western blot analysis confirmed declined secretion of not only full-length S protein, but also L and M proteins (Figure 6B). No gp19/p16 was secreted in such co-transfection experiments. It remains to be determined whether the inhibition of HBsAg secretion was mediated by pg19/p16 retention of L protein, or if N65-tS+ could inhibit HBsAg secretion from N65, which expresses only full-length S protein. Also to be determined is whether virion secretion from the 1.1 mer construct is inhibited. In this regard a previous work found that the W172* nonsense mutation, removing C-terminal 55 residues from S protein, blocked secretion of both virions and SVPs [33]. This W172* mutant showed some dominant negative effect on virion secretion from the WT clone, although the truncated S protein became secreted in the presence of full-length S protein. We recently found that the L209* and W201* nonsense mutations as well as the FS189 frameshift mutation in the S protein could also block HBsAg secretion [22]. Moreover, an R169P substitution in the S protein could inhibit the secretion of both virions and SVPs and had dominant negative effect on virion and SVP secretion from the WT construct [34]. Considering that blocking virion secretion can prevent HBV spread inside the liver, while blocking SVP secretion may promote HBsAg seroconversion, the therapeutic goal, further characterization of the dominant negative effects of such S protein mutants will have important therapeutic implications.

## Figures and Tables

**Table 1:** Point mutations introduced to N51 to prevent expression of L/M proteins or S. protein, or to affect expression of N-terminally truncated S proteins *.

N51	nt **ATG**………………**ATG**……TCG…**ATG**…………CGCTGGATGTGTCTG………………………TAA aa M M S M R W M C L *
N65	nt **ATG**………………**ATG**……**TAG**…**ATG**…………CGCTGGATGTGTCTG………………………TAA aa *
N65-tS	nt **ATG**………………**ATG**……**TAG**…GCG…………CGCTGG**ATG**TGTCTG………………………TAA aa * A
N65-tS+	nt **ATG**………………**ATG**……**TAG**…GCG…………CGCAGG**ATG**GGTCTG………………………TAA aa * A R G
N65-nS	nt **ATG**………………**ATG**……**TAG**…GCG…………CGCTGGGCGTGTCTG………………………TAA aa * A A
N67	nt **ATG**………………**ATG**……TCG…GCG…………CGCTGG**ATG**TGTCTG………………………TAA aa A
N67+	nt **ATG**………………**ATG**……TCG…GCG…………CGCAGG**ATG**GGTCTG………………………TAA aa A R G
N67-ns	nt **ATG**………………**ATG**……TCG…GCG…………CGCTGGGCGTGTCTG………………………TAA aa A A
N67a	nt **ATG**………………**ATG**……TCG…ATA…………CGCTGG**ATG**TGTCTG………………………TAA aa I
N67a+1	nt **ATG**………………**ATG**……TCG…ATA…………CGCGGG**ATG**GGTCTG………………………TAA aa I G G
N67a+2	nt **ATG**………………**ATG**……TCG…ATA…………CGCAGG**ATG**GGTCTG………………………TAA aa I R G
N67a-	nt **ATG**………………**ATG**……TCG…ATA…………CGTTTT**ATG**TGTCTT………………………TAA aa I F
N67a-nS	nt **ATG**………………**ATG**……TCG…ATA…………CGCTGGGCGTGTCTG………………………TAA aa I A
N67b	nt **ATG**………………**ATG**……TCG…AAG…………CGCTGG**ATG**TGTCTG………………………TAA aa K
N67b+1	nt **ATG**………………**ATG**……TCG…AAG…………CGCGGG**ATG**GGTCTG………………………TAA aa K G G
N67b+2	nt **ATG**………………**ATG**……TCG…AAG…………CGCAGG**ATG**GGTCTG………………………TAA aa K R G
N67b-	nt **ATG**………………**ATG**……TCG…AAG…………CGTTTT**ATG**TGTCTT………………………TAA aa K F
N67b-nS	nt **ATG**………………**ATG**……TCG…AAG…………CGCTGGGCGTGTCTG………………………TAA aa K A
N67c	nt **ATG**………………**ATG**……TCG…TTG…………CGCTGG**ATG**TGTCTG………………………TAA aa L

* The four ATG codons in N51 are the preS1, preS2, S start codons and codon 75 of the S region, respectively. Shown in bold are translation start or stop sites for each construct. Shown in red are mutated nucleotides or amino acid (aa) relative to N51.

**Table 2:** Mutations in 1.1 mer construct of genotype A or D to prevent core, L, or S protein expression or to affect a preS2 neutralizing epitope.

Mutation	Genotype (Clone)	Change (nt)	Change (aa)	Change (aa, Overlapping Gene)
**core**	A (geno5.4)	C2044A	Core: C48*	N/A
**core**	D (geno1.2)	T2044A	Core: C48*	N/A
**L**	A (geno5.4)	G3008A	L: W52*	Pol: silent
**L**	D (geno1.2)	A2959T	L: K38*	Pol: Q218L
**S**	A (geno5.4)	T156C	S: M1T	Pol: silent
**S**	D (geno1.2)	T156C	S: M1T	Pol: silent
**S**	D (#)	T156C	S: M1T	Pol: silent
**AAAA** **(preS2)**	D (#)	##	preS2: Q13A/D14A/P15A/R16A	Pol: A301G/Q304G
**del13–16** **(preS2)**	D (#)	del^26^CAAGATCCCAGA^37^	preS2: del^13^QDPR^16^	Pol: del^301^ARSQ^304^/S305G

#: [18]. ##: C26G/A27C/A28C/A30C/T31A/C32G/C34G/A35G/G36C.

## Data Availability

Original data are available in notebooks, blots, computer, etc.
